# Identifying subgroups of teacher burnout in elementary and secondary schools: the effects of teacher experience, age and gender

**DOI:** 10.3389/fpsyg.2024.1406562

**Published:** 2025-01-13

**Authors:** Mohammed H. Alghamdi, Georgios Sideridis

**Affiliations:** ^1^Department of Self-Development Skills, King Saud University, Riyadh, Saudi Arabia; ^2^Department of Primary Education, National and Kapodistrian University of Athens, Athens, Greece

**Keywords:** teacher burnout, TIMSS 2019, Saudi Arabia, elementary education, Secondary education, teacher experience, teacher’s gender, latent class analysis

## Abstract

**Introduction:**

Teacher burnout is a serious problem that requires quick attention and management since it not only compromises educational quality but also strains schools’ financial resources.

**Research objective:**

The purpose of the present study was to profile burnout indicators for teachers in the Kingdom of Saudi Arabia. A secondary goal was to evaluate the consistency of burnout profiles between elementary and secondary school teachers.

**Method:**

Participants were 703 teachers who participated in the Trends in International Mathematics and Science Study (TIMSS 2019) and were part of the measurement in Saudi Arabia and participated in this study. A series of nested latent class models were run using 1–6 classes to identify an optimal number of interpretable subgroups (latent classes) that best describe the latent construct of teacher burnout in the elementary and high school setting.

**Results:**

The most important finding was that more than one-third of the teachers reported high levels of burnout irrespective of education level or gender. Furthermore, teachers’ experience and age were positive predictors of burnout with older teachers having significantly elevated levels of burnout. Gender on the other hand did not play a significant moderating role in teachers’ levels of burnout.

**Implications:**

Reducing the number of students in each class, reorganizing the administrative work that has to be done, adding more support personnel to classrooms, and increasing time management skills and tactics via professional development programs are all potential solutions that might help alleviate the problem of teacher burnout.

## 1 Introduction

Due to its potential negative effects on student progress and the school system as a whole, teacher burnout is a serious problem. Significant negative links between teacher burnout and teacher performance have been consistently reported with concomitant effects on students’ lowered achievement gains (e.g., Grayson and Alvarez, 2008). This negative effect is likely linked to the teacher’s inability to provide an ideal learning environment for students to develop and flourish as an overworked teacher’s stress may be perceived by students. Further insights on the effects of burnout at the school level have been introduced by Skaalvik and Skaalvik (2017) who reported a link between burnout and attrition, creating additional stress for the school system in hiring and training new personnel. Consequently, the effects of teacher burnout are multifaceted as they negatively affect both student morale and achievement as well as the school’s functioning by straining a school’s financial resources. At the personal level, direct links have also been observed between experiences of burnout and physical and emotional health for teachers (Kyriacou, 2001). Mental health symptoms include depression, anxiety, and other stress-related diseases (Aloe et al., 2014). Additionally, it disturbs the learning environment and hurts the morale of other instructors (Brock and Grady, 2000). Therefore, school policies need to prevent teacher burnout by continuously monitoring workloads, offering support and professional development, and providing incentives. This is proposed not only as a means to maintain the quality of instruction and the financial security of the schools but also to ensure the physical and mental health of the teachers who are the most significant resource for students’ education and development outside the home.

Levels of burnout in the international literature have been worrisome, to say the least. In Europe, for example, rates among Dutch teachers have been reported around 20% (Schaufeli et al., 2009) for any form of burnout, and the same rate was observed in the UK (National Foundation for Educational Research, 2017), with comparative rates in other professions being approximately 13%, representing a 35% increase in reported burnout. In the U.S., rates have been elevated with 58% of the teachers reporting mental health problems (American Federation of Teachers, 2017) and about 50% of them reporting high stress (Gallup, Inc., 2014). Alarming rates were observed in Canadian teachers with 90% of them reporting that their job has been stressful (Canadian Teachers' Federation, 2011). In China, elementary and secondary school teachers reported “compassion fatigue” 44% of the time (Yu et al., 2022). As if these rates are not worrisome, the effects of the pandemic have exacerbated levels of stress and anxiety in teachers (Chang et al., 2022), thus, magnifying the problem and its potential consequences.

According to some experts, burnout is the result of years of high workloads and an inability to handle the demands of the job (Maslach et al., 2001). There are several consequences of teacher burnout, personal and professional. On a personal level, burnout is linked to emotional exhaustion (Mamo, 2022; Tsouloupas et al., 2010), increased irritability (Capone et al., 2019), low motivation and autonomy (Alibakhshi et al., 2020; Fernet et al., 2012), poor well-being (Lauermann and König, 2016). In the workplace, teacher burnout has been associated with ineffective teaching strategies to deal with difficult classroom behaviors (Lamude and Scudder, 1992), teacher absenteeism (Schonfeld, 2001), teacher turnover (Marvel et al., 2006; Whipp et al., 2007), or intention to quit (Klassen and Chiu, 2011). Students’ lack of motivation and autonomy, apathy, aberrant behaviors, confrontations with teachers, and poor academic performance are all related to teacher burnout (Grayson and Alvarez, 2008; Tschannen-Moran and Woolfolk Hoy, 2007).

### 1.1 Teacher burnout: context and the roles of teaching experience, age, and gender

Saudi Arabia’s education system, over the past two decades, has been rapidly expanding and undergoing reform such as having more student enrollment, international students, and new curricula that are in alignment with the goals of Vision 2030. These factors probably magnified teacher workloads, which increased in response to changing educational standards and technologies. Saudi schools are major sites of cultural production, and teaching in Saudi is a stressful job as state and society expect teachers to be models of cultural and religious norms. To make matters worse, the top-down administration of the educational sphere can only make burnout symptoms more acute by its regulations- limited teacher autonomy is part of that centralized system. On the other hand, due to its tribal and collectivist social nature (which may offer certain teachers a strong emotional and social safety net), burnout might be lower in some subgroups. Considering the cultural and systemic influences found in this study can help explain our results, showing how interventions may be better focused to align with the specific challenges faced by teachers in Saudi Arabia.

Teacher burnout and teacher experience are two interconnected areas that have been the subject of much educational research with conflicting results (Dias et al., 2021; Gavish and Friedman, 2010). A positive association has indicated that teachers with more experience are likely to be exhausted and overworked, thus, reporting higher burnout levels (Prasojo et al., 2020; Kimsesiz, 2019). An inverse association between experience and burnout suggests that experience leads to higher resilience and efficient management and coping strategies, thus having a negative relationship with burnout (Mousavy et al., 2012). The two potential directions are also evident with the role of age as the correlation between age and teaching experience is positive and strong given the motto that “with age comes experience.” Regarding gender, conflicting results have also been observed as several researchers confirmed the null effects of gender on burnout (Chang, 2009; Kreuzfeld and Seibt, 2022; Răducu and Stănculescu, 2022). Others found that male teachers report higher levels of burnout compared to female teachers (Atashpanjeh et al., 2020) or the exact opposite, that female teachers report higher burnout levels compared to male teachers (Purvanova et al., 2010; Stengård et al., 2022; Capone et al., 2019; Redondo-Florez et al., 2020). Thus, with regard to the roles of teaching experience, age, and gender, on teacher burnout, the jury is still out.

### 1.2 Theoretical links, novelty and importance of the present study

Based on the Job Demands-Resources model (JD-R), occupational stress and in its extreme form, burnout, are a function of the interplay between job demands and the availability of resources (Bakker and Demerouti, 2007, 2017; Demerouti et al., 2001). The job demands represent physical, psychological, social or organizational aspects of a job that require sustained effort or skill and are therefore associated with certain physiological and /or psychological costs (Schaufeli et al., 2009). Examples include high pressure and large workloads. Job resources on the other hand represent “tools” to accommodate word demands and achieve goals and targets. Examples include reducing unrelated to teaching tasks such as administrative work, have access to training and development, reduce class size, etc. Our person-based analyses will contribute to identifying distinct and latent subgroups of teachers that describe the interplay between job demands and resources. For example, it is likely that the observation of a high burnout group displays an imbalance between demands and resources with the former outweighing the latter. On the other hand, a low-burnout group may point to sufficiency of resources in light of the job demands. Besides resources, variables that can act as a buffer to burnout can be experience and age with older and more experienced teachers being more efficacious and competent, or the opposite (younger teachers being newly trained and more adept to technological advances).

The purpose of the present study was to profile burnout indicators for teachers in the Kingdom of Saudi Arabia. A secondary goal was to evaluate the consistency of burnout profiles between elementary and secondary school teachers. A third goal was to evaluate the role of teaching experience in profiling burnout.

## 2 Method

### 2.1 Participants and Procedures

Participants were 703 teachers who participated in the TIMSS 2019 and were part of the measurement in Saudi Arabia (Mullis et al., 2019). There were 335 4th-grade teachers and 368 8th-grade teachers. Amongst the 8th-grade teachers, there were 184 teachers of math and 184 teachers of science. The average experience was 12.55 years (SD = 7.895). There were 345 females and 349 males (gender information was missing from 9 participants). The distribution of age was as follows: 0.9% under 25 years, 11.3% 25–29 years, 48.7% between 30 and 39 years, 31.5% between 40 and 49 years, and 7.6% between 50 and 59 years (*n* = 5 missing data cases). Most teachers had post-secondary education degrees such as a bachelor’s degree (93.9%). Graduate education in the form of a master’s or doctorate degree was attained by 3.4% of the sample. Only 2.8% did not have formal education beyond secondary education. All methods carried out in the present study were following relevant guidelines and regulations specifically the Declaration of Helsinki and were run under the TIMSS 2019 international study protocols and procedures. Regarding sampling, the TIMSS scientific team engages a stratified multistage sampling methodology to ensure population representation across strata such as schools, regions, and SES. A three-step process involves stratifying schools across regions and SES, randomly selecting schools from each stratum, followed by randomly selecting teachers from the chosen schools. Participants completed written consent forms before participating in the study as per TIMSS guidelines. More detailed information on the methodology utilized in TIMSS can be found in this source.^1^

### 2.2 Measures

#### 2.2.1 Teacher burnout

As a measure of teacher burnout, an 8-item scale from the TIMMS 2019 data with the name “About being a Teacher” was used (see Appendix). According to Maslach (1982), the factors that contributed to burnout were the number of pupils, the workload, the lack of time, and the teacher’s perception of pressure. Higher scores indicated lower levels of teacher burnout since the items were scored on a 4-point scale from “Agree a lot” to “Disagree a lot.” However, we reversed the scaling so that higher scores would be indicative of high levels of teacher burnout. Internal consistency reliability with the current data was 0.785 using Cronbach’s alpha and 0.790 using the omega coefficient, both being acceptable. More on the validity of the measure using Item Response Theory (IRT) has been thoroughly described here: https://timssandpirls.bc.edu/timss2019/methods/chapter-16.html.

#### 2.2.2 Teacher experience and age

Teacher experience was a continuous variable assessed over the years. The teacher’s age was assessed using an ordinal scaling category system with the following categories: 1 = Under 25; 2 = 25-29, 3 = 30-39, 4 = 40-49; 5 = 50-59; and 6 = 60 or more.

### 2.3 Data analyses

#### 2.3.1 Latent class analysis: description and model estimation

Latent Class Analysis (LCA) is part of structural equation modeling, with the goal of deciphering classes that are not observed, but rather inferred from the data (Collins and Flaherty, 2002; Collins and Graham, 2002; Vermunt, 2008). According to their patterns of responses to several markers defining one or more latent variables, people are classified into latent classes in LCA (Goodman, 1974). The objective is to identify the fewest classes possible that may correctly explain patterns in the observed variables (Collins, 2006; Collins and Lanza, 2010; Henry and Muthen, 2010). The underlying, unobserved categorical variable that categorizes individuals into separate groups or classes is assumed to be the root cause of the observed response patterns in latent class analysis (Lazarsfeld and Henry, 1968). LCA has one benefit over conventional clustering methods: it offers a formal statistical model that takes into consideration the probabilistic nature of class assignments (Lubke and Muthén, 2005, 2007). As a result, the number of classes may be statistically tested, and variables and distant outcomes can be included in the model (Nylund et al., 2007). The fact that LCA offers class membership probability for each person in the data is another significant component of the method. This acknowledges that categorization is not always simple and allows for ambiguity in class membership (Magidson and Vermunt, 2002).

One of the most critical aspects of LCA is to produce accurate, valid, and parsimonious subgroups that are reflective of true subpopulations. This is why the class enumeration process is of critical importance. One important part of the enumeration process is the guidance provided by information criteria to identify an optimal number of subgroups using the principle of parsimony (Banfield and Raftery, 1993; Raftery, 1995). In the present study, we utilized the AIC, the BIC, and its sample-adjusted variant, the Modified AIC (MAIC, Bozdogan, 1987) for which lower values are indicative of better model fit (Cavanaugh, 1997). The Akaike Information Criterion (AIC) engages estimates of the loglikelihood and the number of estimated parameters but is considered too liberal in that it does not sufficiently penalize additional complexity. As a better alternative, the Bayesian Information Criterion (BIC, Schwarz, 1978) has been recommended as providing a more appropriate penalty and so does the modified AIC both being appropriate for relatively small sample sizes, providing an appropriate penalty for that purpose (MAIC, Bozdogan, 1987). Another interesting criterion put forth by Masyn (2013) is the correct model probability index (cmP) which is valid assuming one of the tested models is the true model in the population.

#### 2.3.2 Model classification accuracy

Two indices were selected to evaluate classification accuracy and thus latent class homogeneity. These were (a) the average posterior probabilities (AvePP), and (b) the Odds of Correct Classification (OCC) (Nagin, 2005; Masyn, 2013). The AvePP provides an overall index of the model’s classification accuracy (Asparouhov and Muthén, 2014). Estimates range between 0 and 1, with larger values being indicative of greater certainty in the classification of persons into subgroups. Estimates greater than 0.80 are indicative of good model separation, and confidence in assigning individuals to their most likely latent class based on their response patterns to the indicators of a latent variable (Rost, 2006).

The Odds of Correct Classification (OCC) is the ratio of the odds of classification into a latent class based on the maximum posterior probability compared to the odds derived from random classification (Weller et al., 2020). If this ratio is greater than 1, it means that the Maximum Posterior Probability classification rule is doing better than random guessing. If it’s less than 1, the Maximum Posterior Probability classification rule does worse than random guessing. Estimates greater than 5 signal good separation (Nagin, 2005).

Additionally, entropy, although not valued in the class enumeration process, provided evidence for proper class formation, accuracy, and the ability of the model to identify distinct, rather than overlapping subgroups. In other words, entropy shows the amount of certainty with which participants are assigned to groups with higher numbers showing confidence in classifications (Celeux and Soromenho, 1996). Based on Clark and Muthén (2009) entropy estimates close to 0.70 or greater are indicative of a strong association of individuals with a specific group.

#### 2.3.3 LCA invariance by educational level

Latent class equivalence was assessed using a 3-step approach so that the consistency between latent groups in elementary versus secondary education will be confirmed. The procedure involves estimating a multi-group unconstrained model (Model A), a semi-constrained model (Model B), and a fully constrained model (Model C). The first model estimates the latent classes for the grouping variable (elementary/secondary in our case) with freely estimated thresholds as well as class sizes per group. This model is contrasted to Model B which sets item thresholds to be equivalent across 4th and 8th grade teachers but with still the class sizes allowed to vary freely across groups. Model B is then contrasted to Model C, which constrains both item intercepts and class sizes to be equivalent across levels of education, elementary and secondary. Model comparison is conducted solely using information criteria with the most preferred being the BIC and its sample-adjusted variant (SABIC), as the likelihood ratio test is not available in the context of multilevel modeling (i.e., the presence of clustering due to schools).

#### 2.3.4 LCA sample size and power estimation

Power in the LCA model targeted at being able to identify 6 distinct classes using 8 measured variables as in the case of teacher burnout. Subgroups were posited to have −2, −1, 0, 0.5, 1, and 2 mean values in logits across all 8 binary predictors, pointing to ideal separation and within-class homogeneity. Using a proposed sample size of *n* = 700 teachers a Monte Carlo simulation was run in Mplus 8.10 (Muthén and Muthén, 1998-2017). Coverage across 1,000 replicated samples ranged between 91.6 and 96%, pointing to good parameter recovery. The mean loglikelihood value across 1,000 replicated samples was 18077.264 compared to an expected value of 18076.030. Rules of thumb on sample sizes in latent class analysis pointed to *n* > 500 (Finch and Bronk, 2011; Nylund-Gibson and Choi, 2018), which agrees with the results from the present simulation.

## 3 Results

### 3.1 Subgroups of teacher’s burnout experiences

**Figure 1:**
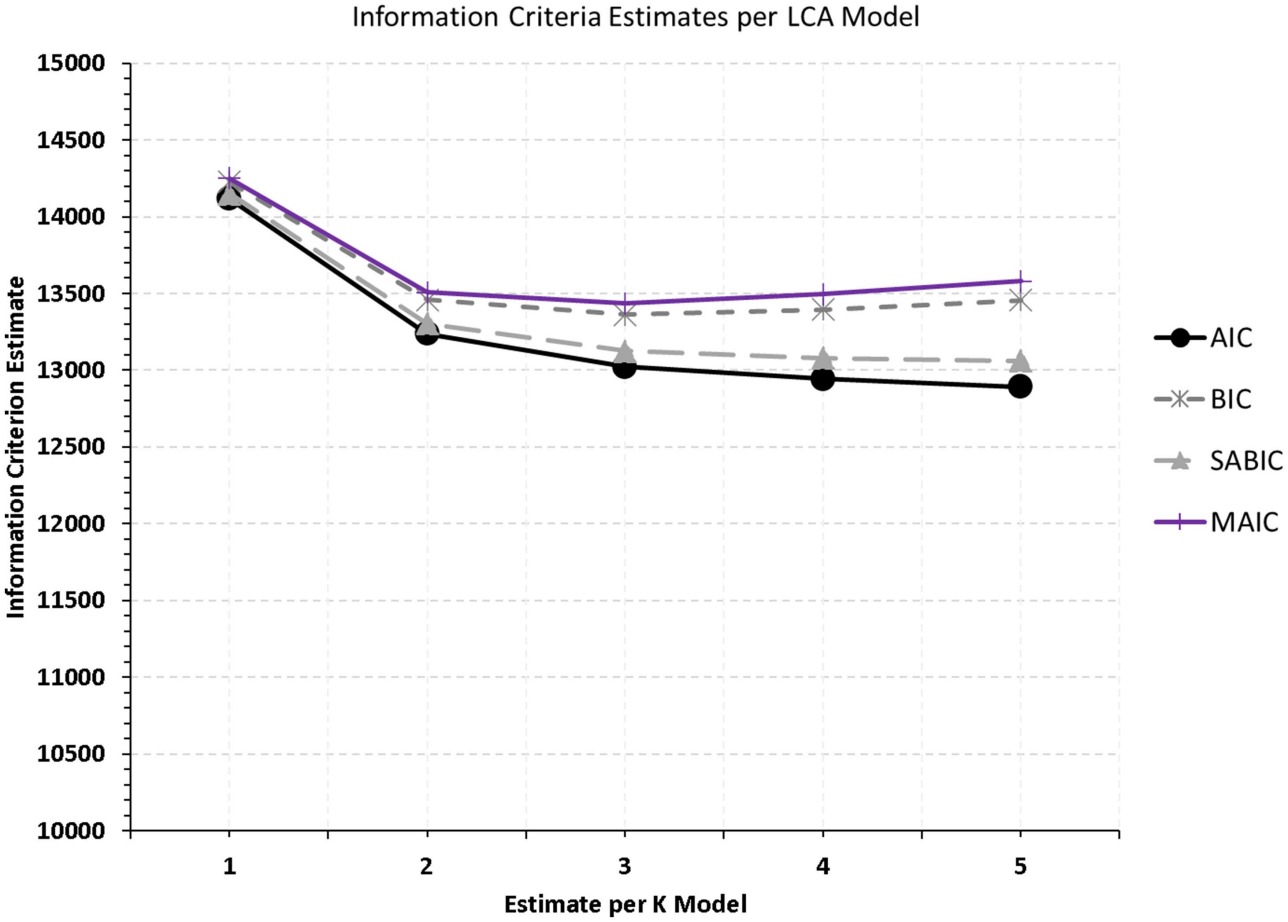
Elbow plot examining optimal subgrouping of teacher burnout experiences in Saudi Arabia.

A series of nested latent class models were run using 1-6 classes to identify an optimal number of interpretable subgroups that best describe the latent construct of teacher burnout in the elementary and high school setting in Saudi Arabia. As shown in Table 1, among information criteria, a 3-class solution was favored by the BIC, the MAIC, and the cmP for model ‘k’. As expected, the AIC favored more subgroups confirming the lack of penalty as the number of estimated parameters increased and so did the sample-adjusted BIC which showed a preference for 5 classes. Figure 1 displays elbow plots with values of the information criteria used per latent class model. As shown in the figure, there is a negative trend (indicating improvement from adding classes), up to the measurement of three latent subgroups. A fourth subgroup was not supported by the BIC, and the MAIC for which estimates increased pointing to a worse model fit and a loss in parsimony. Consequently, the elbow plot of Figure 1 suggests that the most appropriate solution is inclusive of three latent groups. Values of entropy, although not contributing to latent class decision-making, showed that the 3-class model showed increased homogeneity with a value of 0.735, greater than the cutoff of 0.700 suggested earlier (Depaoli, 2012).

**Table 1:** Model fit for 1–6 classes when profiling teacher burnout experiences in Saudi Arabia.

Model tested	LL	Npar	AIC	BIC	SABIC	MAIC	cmP(k)	Entropy
1-class	−7034.21	24	14116.42	14225.75	14149.54	14249.75	0.00	–
2-class	−6569.04	49	13236.07	13459.28	13303.70	13508.28	0.00	0.787
3-class	−6437.63	74	13023.27	**13360.36**	13125.40	**13434.36**	**1.00**	0.735
4-class	−6372.97	99	12943.94	13394.92	13080.58	13493.92	0.00	0.729
5-class	−6321.25	124	12890.50	13455.36	13061.63	13579.36	0.00	0.743
6-class	−6278.94	149	12855.88	13534.63	13061.52	13683.63	0.00	0.768

LL, Loglikelihood; Npar, number of estimated parameters; AIC, Akaike Information Criterion; BIC, Bayesian Information Criterion; SABIC, Sample adjusted BIC; MAIC, modified AIC; cmP(k), correct model probability for model k. Bold values indicate preferred model.

### 3.2 Latent class classification accuracy and interpretation

**Figure 2:**
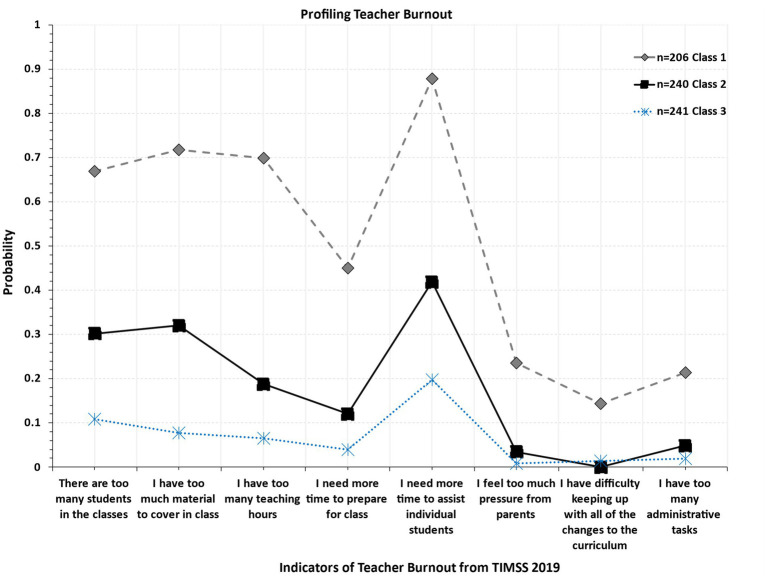
Optimal latent class solution when profiling teacher burnout in Saudi Arabia.

Table 2 displays information on classification accuracy. As shown in the table, the Average Posterior Probability of Classification (AvePP) was equal to 0.925 (greater than a cutoff of 0.70 set forth by Nagin, 2005). Furthermore, estimates for the Odds of Correct Classification (OCC) were equal to 23.006 (greater than the cutoff recommendations of 5 units). Thus, collectively, the three latent groups that emerged were highly homogeneous but also distinct from each other. Figure 2 shows the 3-class model showing a highly burned-out group comprising 30% of the sample (Class 1). Of particular concern was the lack of time and the need to allow more time for assisting students. Too much teaching and material to cover and in the presence of many students were the ultimate concerns for these teachers. Of less concern were content issues related to time pressures and potential difficulties with keeping up with the curriculum or having too many administrative tasks. A low to medium burned-out class (Class 2) was represented by 35% of the sample for which the only behavior that was more prevalent was again related to the need to have more time to assist students. Last, the low burned-out class comprised 35% of the sample and had low levels throughout the scale items.

**Table 2:** Classification accuracy and separation for optimal 3-class solution.

Class number	Estimated proportion	AvePP	OCC
Class 1	36.8%	0.844	9.292
Class 2	28.3%	0.868	16.660
Class 3	34.9%	0.925	23.006

Estimated proportion reflects the class size as a function of the total sample size; AvePP, average posterior probabilities; OCC, odds of correct classification.

### 3.3 Measurement invariance of latent classes across elementary and secondary school teachers

**Figure 3:**
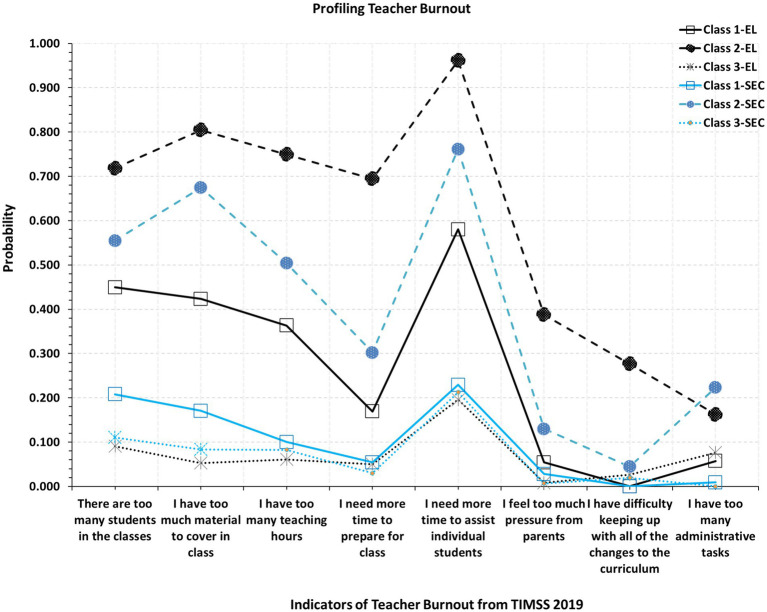
Profiling teacher burnout across elementary and secondary school settings in Saudi Arabia.

A secondary hypothesis pertained to testing whether the observed latent subgroups were equivalent in both form and representation between 4th-grade and 8th-grade teachers. For that purpose, we tested three consecutive models as described above. As shown in Table 3, except for the AIC, both BIC and SABIC pointed to the superiority of Model C, which suggests equivalence of class thresholds for each latent subgroup and also the proportions of class manifestation in both elementary and secondary school settings. Figure 3 displays the solution for each of the two levels of education. Despite supporting equivalence, it is apparent from Figure 3, that levels of burnout were elevated across almost all behaviors for elementary school teachers, compared to secondary school teachers, but, that difference was within expected, random measurement error.

**Table 3:** LCA measurement equivalence in elementary vs. secondary school settings.

Model tested	AIC	BIC	SABIC
Model A (Unconstrained)	**15197.58**	15475.03	15281.35
Model B (Semi-Constrained)	15214.19	15419.19	15276.30
Model C (Fully Constrained)	15215.25	**15411.13**	**15274.59**

Bold entries indicate the preferred model by criterion value.

### 3.4 Predicting teacher burnout from teaching experience, age, and gender

**Figure 4:**
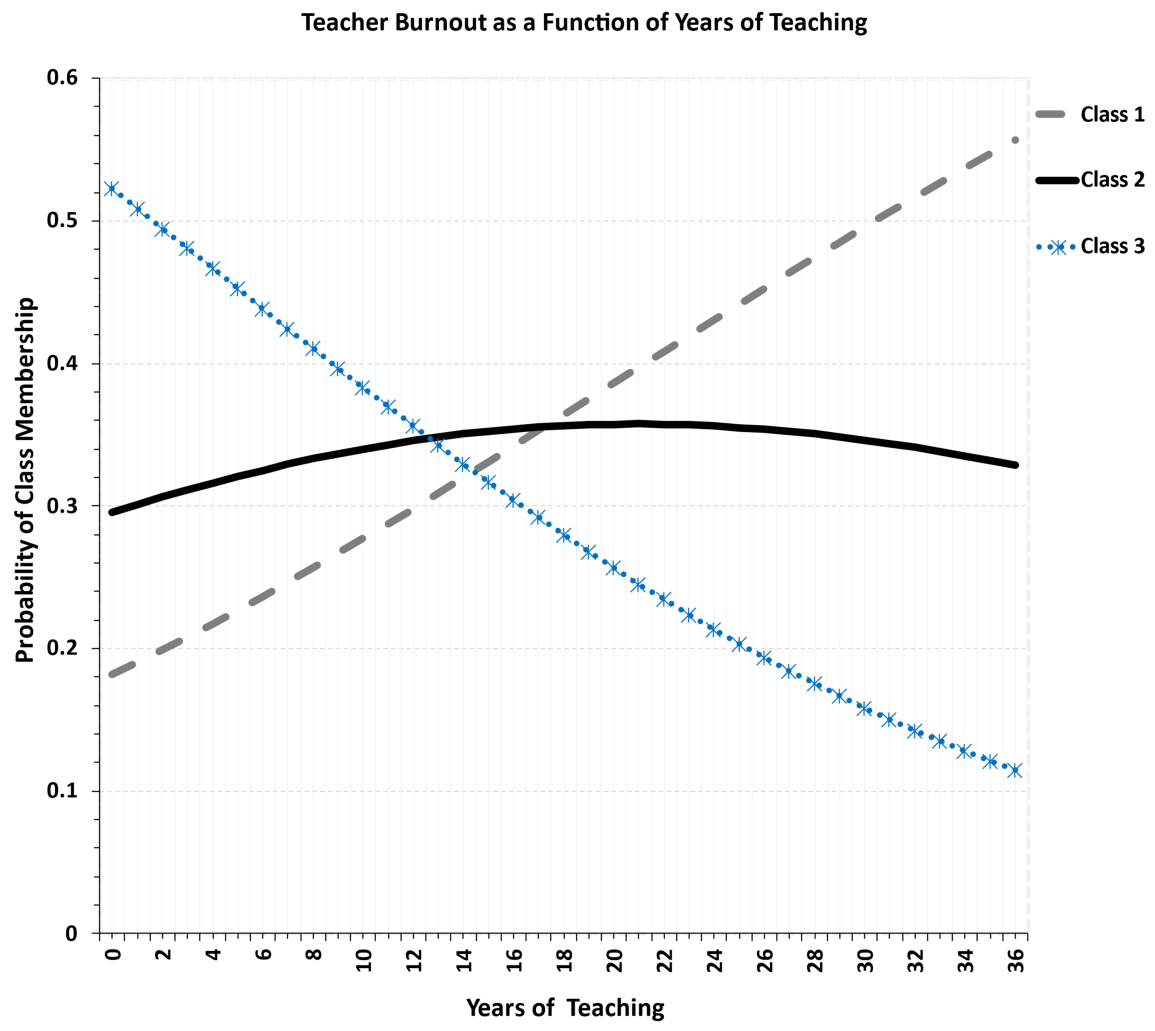
Relationship between teacher years of experience and latent class membership.

The moderating effect of teaching experience on burnout is depicted in Figure 4. Starting with class 3, the low-burnout class, the probability of being a member of this class lowers as years of teaching experience increase. Thus, latent class 3 is defined by an inexperienced, likely younger group of teachers, for which potentially negative experiences have not piled up to create a sense of burnout. Class 1, the high burnout group held a positive relationship with teaching experience. In other words, more experienced teachers were more likely to belong to the high burnout class. Last, for Class 2, the low-to-medium burnout teacher group, teaching experience did not play a significant role, pointing to the null effects of experience. The probability of belonging to the high burnout class was equal across almost all age groups. That is, although a positive quadratic trend was observed, this was within the margin of measurement error. That is, teachers in the high burnout group had from very few to very many years of teaching experience. Statistically speaking the trajectory of teaching experience was significantly more positive in class 1, the lowo-burnout class, compared to class 3 (*b* = 0.073, *p* < 0.001) and class 2 compared to class 3 (*b* = 0.045, *p* = 0.014).

**Figure 5:**
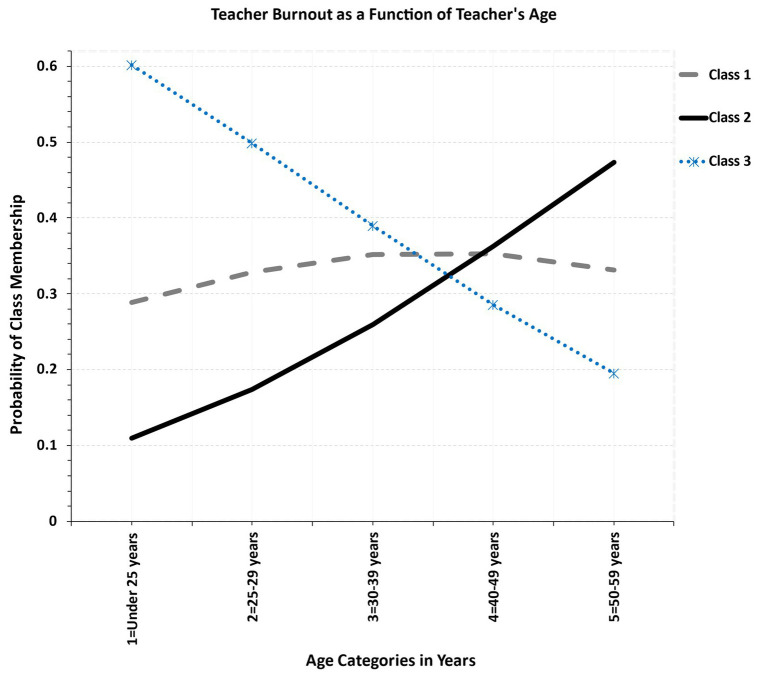
Relationship between teacher’s age in years and latent class membership.

Given the strong relationship between age and teaching experience (*r* = 0.749, *p* < 0.001), there was a strong resemblance between the respective findings of age and years of experience (Figure 5), and are thus the interpretations described above hold for age as well. Briefly, the high burnout class was associated with older teachers whereas the low burnout class of younger teachers. For the low to medium burnout class, gender was distributed approximately equally.

**Figure 6:**
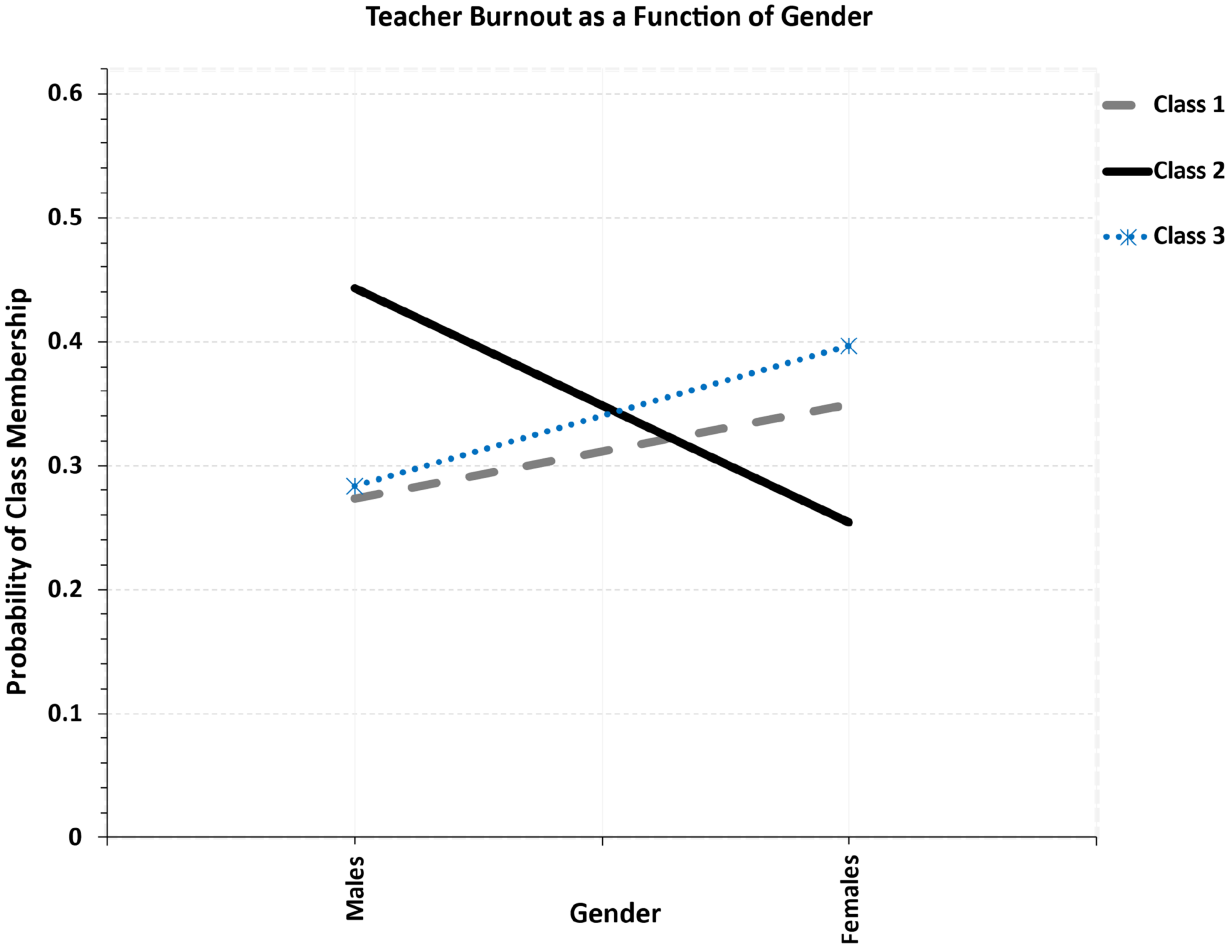
Relationship between teacher’s gender and latent class membership.

For gender, the high burnout class was equally balanced across genders (49.2% males, 50.8% females). Using multinomial regression, differences were evident as the low burnout class (class 3) had more females (55.2%) compared to the low-to-medium burnout class (class 2) that had significantly more males (56.2%) with the difference being significant (z-test = 2.011, *p* = 0.044). There were no significant between classes differences on gender across other combinations of classes (see Figure 6).

## 4 Discussion

The purpose of the present study was to profile burnout indicators for teachers in the Kingdom of Saudi Arabia. A secondary goal was to evaluate the consistency of burnout profiles between elementary and secondary school teachers. A third goal was to evaluate the role of teaching experience in profiling burnout. The most important finding was that more than one-third of the teachers reported high levels of burnout. These rates have been larger compared to studies in the Netherlands, Turkey, Northern Cyprus, and China (i.e., less than 25%, Ozan, 2009; Yu et al., 2022) and lower compared to the United States (44%) and Canada (25-74%).

Furthermore, the most important concern for teachers has been the lack of time, which may be particularly crucial for the provision of student quality experiences, individualized attention, and support. A student’s educational career is crucially dependent on individualized attention. It enables instructors to pinpoint each student’s unique strengths and weaknesses, accommodate various learning preferences, and provide individualized help and feedback. However, when teachers are overworked by meetings, administrative work, big class numbers, and other duties, they are less able to provide each student with the customized attention that is so important, especially in the presence of learning challenges. When teachers are overworked, they may resort to one-size-fits-all teaching strategies that ignore the unique needs of each student (Konstantopoulos and Chung, 2011).

The second most important concern has been a reported overload about having too much teaching, too much material to cover, and the presence of large classes and a small teacher-to-student ratio. These concerns likely arise from the introduction of the educational reform as posited in Vision 2030 as knowledge on new curricula and instructional strategies is mandated at limited timeframe. Among those pressures is also the need for students to catch up with international benchmarks such as those from PISA, TIMSS, PIRLS, TALIS, and other international studies. Based on Skaalvik and Skaalvik (2010) the pressure to meet these expectations may result in a loss of self-esteem, feelings of inadequacy, and frustration, thus likely compromising teaching quality. Furthermore, teachers may feel undervalued and overwhelmed and may exhibit internalized symptoms such as anxiety (Aloe et al., 2014; Dicke et al., 2018).

Teaching experience and its correlate teacher’s age played a significant role in teachers’ burnout experiences and because of their strong relationship will be described together. The main finding was that high levels of burnout were observed for older, albeit more experienced teachers (class 1), and the opposite was observed for the low burnout class (class 3) which was primarily comprised of younger teachers. The high burnout levels in older teachers may be explained by the rapid technological advances and educational practices that move beyond traditional teacher-centered models. In other words older teachers may be less familiar with developments and may struggle to keep up with technological advances and demands (Gu and Day, 2007). Further explanations may relate to older teachers likely being more physically and cognitively challenged as they may not have the same stamina, motivation, and enthusiasm as they had at the beginning of their careers (Anastasiou and Belios, 2020). Last, older teachers may no longer consider advancements in their careers and have high-end goals for professional growth as do younger teachers (Hargreaves, 2005). The present findings, however, deviate from past studies in which younger teachers reported elevated levels of burnout (e.g., Fantilli and McDougall, 2009).

Gender did not play a significant moderating role in teacher burnout, particularly in the high-burnout class, where male and female teachers were equally represented. This finding suggests that, at high levels of burnout, a set of universal professional stressors may affect both genders similarly. Such stressors may include but are not limited to excessive time demands, increased administrative responsibilities, and the need to allocate time for professional development. As Skaalvik and Skaalvik (2017) reported, time pressures and workload expectations were the strongest predictors of burnout in both male and female teachers. Similarly, Aloe et al. (2014) demonstrated that misbehavior in the classroom and excessive job demands are universal predictors of teacher burnout, irrespective of gender. Within the context of Saudi Arabia, such stressors may be amplified as teachers work with large class sizes and have limited autonomy. Related to the fact that the medium-burnout class had more males, while the low-burnout class had more females, such a finding may reflect differences in gender coping mechanisms or variations in role expectations across gender. For example, studies have shown that male teachers may be less likely to seek emotional comfort and social support (Chang, 2009), which might explain their higher representation in the medium-burnout group. In contrast, female teachers may utilize stronger social networks or prioritize well-being practices, aligning with the cultural norms of caregiving, potentially contributing to their greater representation in the low-burnout class (Capone et al., 2019). Although Saudi Arabia gradually advocates for practices related to gender equality, broader societal expectations may mean that male and female teachers experience burnout differently in their personal and professional lives. Although male and female teachers may experience the same institutional stressors, females may be pressured by societal norms to maintain traditional family roles in addition to their careers and this could to some extent shape their burnout trajectories.

### 4.1 Implications for educational policy and practice

The present study’s findings have important implications for teaching and practice. Reducing the number of students in each class, reorganizing the administrative work that has to be done, adding more support personnel to classrooms, and increasing time management skills and tactics via professional development programs are all potential solutions.

For example, at the top of the list of areas identified as needing major improvement in this study is the overall lack of structure and support that teachers must cope with very large class sizes, especially when dealing with special needs students who desperately require one on one assistance. One solution would be to impose low teacher-to-student ratios in classrooms, especially at schools for which teachers demonstrated high levels of burnout. Administrative tasks were a major factor cited by teachers that was associated with elevated levels in burnout. One potential solution is for schools to employ full-time administrative staff to cope with non-instructional work, classroom assistants, teaching assistants, parent aids, or other support personnel which will in turn allow teachers to concentrate more on coursework planning and teaching efficiency. Targeted professional development programs in time management, priorities, coping strategies etc. would provide teachers with tangible tools they could apply to lower their stress levels. Policymakers could require that teachers attend regularly scheduled training sessions as part of their continuing professional education. Last, in Saudi Arabia, where a collectivist culture is prevalent, there is an opportunity to use community-based support systems as a means of mitigating burnout. Schools can create peer mentoring programs for experienced teachers to help new staff manage workload and stress.

### 4.2 Limitations and future directions

The present study may have been limited for the following reasons. First, the data were collected during 2019, which reflects a 4-year lag from the present day. Second, the sample size was modest, and the study was cross-sectional rather than longitudinal that would allow for the monitoring and prediction of burnout over time. Thus, we recommend replication of the present solution with future datasets that may engage longitudinal designs. Some of the more statistical limitations pertain to the fact that an LCA can produce unreliable solutions if assumptions are violated, if group homogeneity or separation are limited, or when both conditions are operative (Nylund et al., 2007). For example, the presence of local dependency or autocorrelation between indicators may result in latent class over-extraction (Collins and Lanza, 2010). Furthermore, the decision for an optimal model selection reflects some amount of subjectivity as the decision values interpretation and theoretical clarity along with inferential statistical criteria and model interpretation can only be as good as the measured indicators (Collins and Lanza, 2010). Last, the model assigns individuals to classes assuming the emerged categories are exhaustive and mutually exclusive, which is a tentative assumption (Green, 2014). For example, that would most likely not be the case with comorbid groups who may share attributes that are present in more than one class. Despite these potential drawbacks, however, the optimal solution was favored by the most important criteria, interpretation was adequate, and models were run properly with the loglikelihood being replicated with different numbers of starting values.

Future studies may take on two directions. First, there is the need to replicate the teacher burnout profiles that emerged in the present study as being reflective of the population of teachers in Saudi Arabia across both elementary and secondary schools. Second, there is a need to examine the moderating roles of work-related stressors that increase the level of burnout experienced by teachers (Bozkuş, 2018). For example, levels of stress may deviate in general versus special education settings (Dias et al., 2021).

## Figures and Tables

**Figure A1:**
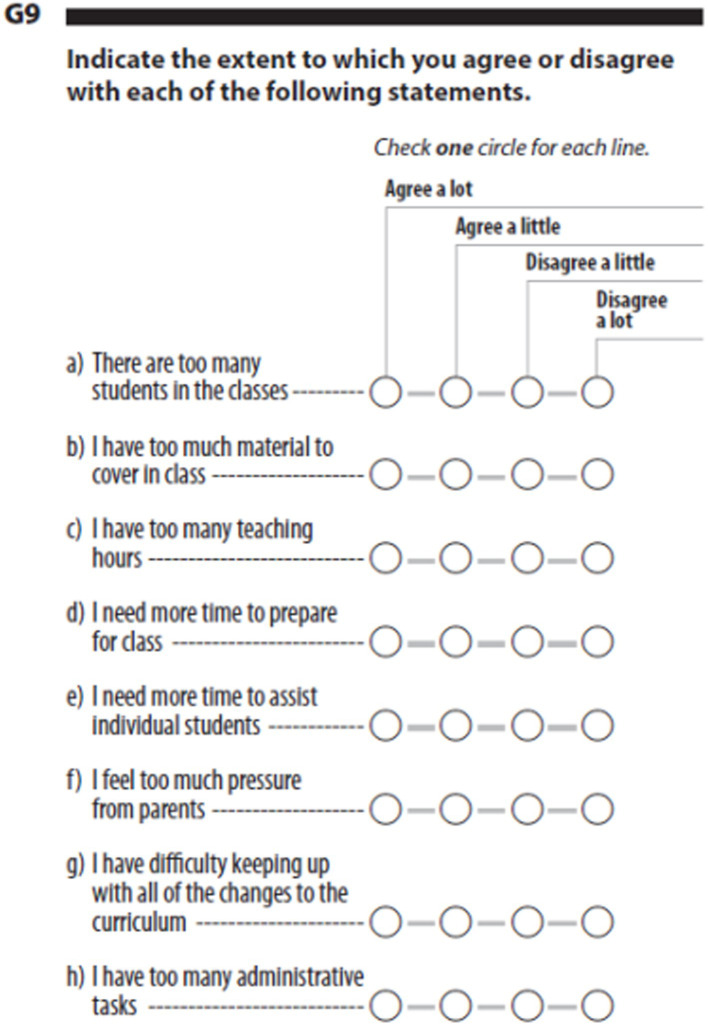
Teacher burnout in TIMSS 2019.

## Data Availability

The datasets presented in this study can be found in online repositories. The names of the repository/repositories and accession number(s) can be found below: https://www.iea.nl/studies/iea/timss/2019.

## Footnotes

^1^ https://timssandpirls.bc.edu/timss2019/
